# Association of nadir serum albumin during hospitalization with mortality in ICU patients with spontaneous intracerebral hemorrhage

**DOI:** 10.3389/fneur.2026.1871576

**Published:** 2026-07-20

**Authors:** Minzhi Zhang, Yang Liu, Zhiying Zhang, Chang Liu, Tao Liu, Chunsheng Yang

**Affiliations:** 1 Department of Neurology, Tianjin Medical University General Hospital, Tianjin, China; 2 Department of Neurosurgery, Tianjin Medical University General Hospital, Tianjin, China; 3 Department of Neurosurgery, Zhangjiakou Second Hospital, Zhangjiakou, Hebei, China; 4 Department of Neurosurgery, Xuanwu Hospital Capital Medical University, Beijing, China; 5 The George Institute for Global Health, University of New South Wales, Sydney, NSW, Australia

**Keywords:** intensive care unit, intracerebral hemorrhage, mechanical ventilation, mortality, serum albumin

## Abstract

**Objective:**

Hypoalbuminemia is common among critically ill patients and has been associated with adverse clinical outcomes. However, the prognostic significance of the nadir serum albumin level during hospitalization in patients with spontaneous intracerebral hemorrhage (ICH) admitted to the intensive care unit (ICU) remains unclear. This study aimed to investigate the association between in-hospital lowest serum albumin levels and clinical outcomes in ICU patients with spontaneous ICH.

**Methods:**

We conducted a retrospective cohort study using pooled data from the Tianjin cohort and Medical Information Mart for Intensive Care IV (MIMIC-IV) database. Adult ICU patients with spontaneous ICH were included. The primary outcome was 28-day all-cause mortality. Multivariable Cox proportional hazards models were used to assess associations between nadir serum albumin during ICU stay and 28-day mortality. Restricted cubic splines (RCS) were applied to examine potential nonlinear relationships.

**Results:**

The incidence of 28-day mortality increased from 17.2% in the >35 g/L group to 39.3% in the < 30 g/L group, while 90-day mortality increased from 21.2% to 52.0% (both *P* < 0.001). Similarly, ICU mortality and in-hospital mortality increased progressively with decreasing albumin levels. In addition, patients with lower albumin levels had significantly longer ICU and hospital stays (both *P* < 0.001). RCS analyses showed no clear evidence of nonlinearity in the fully adjusted model. Subgroup analyses by mechanical ventilation (MV) status showed that lower albumin levels were associated with increased mortality among patients without MV, whereas these associations were attenuated among those receiving MV. Significant interaction by MV status was observed for in-hospital, 28-day, and 90-day mortality (all *P* for interaction < 0.05), but not for ICU mortality.

**Conclusion:**

Lower nadir serum albumin levels during hospitalization are independently associated with increased mortality in ICU patients with spontaneous ICH. The prognostic value of albumin appears to vary by illness severity and treatment intensity, as reflected by MV status. These findings suggest that nadir serum albumin level may be a simple and clinically relevant prognostic marker for risk stratification in this population.

## Introduction

Spontaneous intracerebral hemorrhage (ICH) is among the most devastating subtypes of stroke, characterized by high mortality and substantial neurological disability (1, 2). Neurological injury after spontaneous ICH is not limited to the primary mass effect of the hematoma but also involves secondary brain injury characterized by inflammatory cascades, blood–brain barrier disruption, oxidative stress, and cerebral edema (3–5), processes that may collectively contribute to progressive neuronal damage and neurological deterioration. Despite advances in neurocritical care and intensive monitoring, outcomes among patients with ICH requiring intensive care unit (ICU) admission remain poor (6, 7). Early identification of high-risk patients is therefore essential to guide clinical decision-making and optimize management strategies.

Serum albumin plays a central role in maintaining oncotic pressure, facilitating ligand binding and transport, and exerting antioxidant, anti-inflammatory, and endothelial-protective effects (8, 9). Hypoalbuminemia is common in critically ill patients (10, 11), arising from a combination of increased capillary permeability, systemic inflammation, reduced hepatic synthesis, and nutritional depletion (8, 12). Accumulating evidence indicates that low serum albumin levels are independently associated with adverse outcomes-including increased mortality, prolonged ICU stay, and organ dysfunction-across various critical illnesses, particularly sepsis and acute kidney injury (13–15).

However, most prior studies have focused on albumin levels at admission or on averaged values during hospitalization (12, 14, 15), which may not adequately reflect the dynamic and cumulative physiological derangements occurring during critical illness. The lowest serum albumin level during hospitalization may better capture the integrated burden of inflammation, capillary leakage, and metabolic stress, and thus serve as a more sensitive indicator of disease severity and prognosis (13). To date, the association between the lowest serum albumin level and clinical outcomes in ICU patients with spontaneous ICH has not been systematically evaluated.

Therefore, leveraging data from the Medical Information Mart for Intensive Care IV (MIMIC-IV) database and an independent cohort from the Neurosurgical Intensive Care Unit of Tianjin Medical University General Hospital, we aimed to investigate the association between the lowest serum albumin level during hospitalization and clinical outcomes in patients with spontaneous ICH. We hypothesized that lower albumin nadirs would be independently associated with increased mortality in this population.

## Methods

### Study population

This retrospective cohort study was conducted using data from the Neurosurgical Intensive Care Unit of Tianjin Medical University General Hospital and MIMIC-IV database. Eligible patients from both data sources were combined into a single pooled analytic cohort for the primary analyses.

One author (MZ) obtained access to the database (certification ID: 14,411,525) and performed all data extraction procedures. This study adhered to the ethical standards outlined in the 1964 Declaration of Helsinki and its subsequent amendments. The Ethics Committee of Tianjin Medical University General Hospital approved the study (Approval number: IRB2026-YX-221-01).

Inclusion criteria were as follows: adult patients (≥18 years) with a diagnosis of ICH, identified using International Classification of Diseases codes (ICD-9: 431; ICD-10: I61, I610, I611, I612, I613, I614, I615, I616, I618, I619), who were admitted to the ICU. For patients with multiple ICU admissions, only the first ICU stay was included in the analysis. Exclusion criteria included: hospital length of stay < 24 h; absence of serum albumin measurements during hospitalization; and age < 18 years.

### Patient characteristics

Structured Query Language (SQL) was used to extract relevant clinical data. The following variables were obtained: (1) demographic characteristics, including age, sex, and race; (2) vital signs, including systolic blood pressure (SBP) and diastolic blood pressure (DBP); (3) laboratory measurements, including white blood cell count (WBC), hemoglobin, platelet count, serum creatinine, and blood glucose; (4) comorbidities, identified using International Classification of Diseases, Ninth or Tenth Revision (ICD-9/ICD-10) codes, including myocardial infarction, congestive heart failure, chronic pulmonary disease, renal disease, liver disease, and malignancy; (5) disease severity indicators, including the Charlson Comorbidity Index and Glasgow Coma Scale (GCS) score; and (6) treatment-related variables, including the use of mechanical ventilation (MV). Missing data were limited, with missingness below 5% for all variables included in the primary multivariable regression models. Given the low proportion of missing data, complete-case analyses were performed, and no imputation was conducted.

The primary exposure was the lowest serum albumin level during hospitalization.

To ensure temporal validity, only albumin measurements obtained prior to the occurrence of the outcome event were considered for patients who experienced in-hospital mortality. For patients who survived to discharge, all albumin measurements during hospitalization were included. Furthermore, to minimize the influence of extreme or spurious values, implausible albumin measurements were excluded based on predefined physiological ranges. Patients were categorized into three groups based on clinically relevant thresholds: < 30 g/L, 30–35 g/L, and >35 g/L (reference group).

### Outcome measures

The primary outcome was 28-day all-cause mortality. Secondary outcomes included 90-day all-cause mortality, ICU mortality, in-hospital mortality, length of ICU stay, and length of hospital stay.

### Statistical analyses

Continuous variables are presented as mean (Standard deviation; SD) or median (interquartile range, IQR), as appropriate. Group comparisons were performed using the Student's *t* test or one-way analysis of variance (ANOVA) for normally distributed variables, and the Mann-Whitney *U* test or Kruskal-Wallis test for non-normally distributed variables. Categorical variables are expressed as counts (percentages) and were compared using the Pearson chi-square test or Fisher's exact test, as appropriate.

Logistic regression models were used to analyze ICU mortality and in-hospital mortality, with results reported as odds ratios (ORs) and 95% confidence intervals (CIs). Time-to-event outcomes, including 28-day and 90-day mortality, were analyzed using Cox proportional hazards regression models, with results reported as hazard ratios (HRs) and 95% CIs. Three models with increasing levels of adjustment were constructed: Model 1 was unadjusted; Model 2 was adjusted for age, sex, and race; and Model 3 was further adjusted for SBP, DBP, WBC, hemoglobin, platelet count, serum creatinine, blood glucose, comorbidities (myocardial infarction, congestive heart failure, chronic pulmonary disease, renal disease, liver disease, and malignancy), Charlson Comorbidity Index, and GCS score. The proportional hazards assumption for Cox proportional hazards models was assessed using Schoenfeld residuals.

Given that MV may reflect both disease severity and treatment intensity, subgroup analyses were performed stratified by MV status. Effect modification was assessed by including an interaction term (albumin × MV) in the models, with statistical significance evaluated using likelihood ratio tests. Potential nonlinear associations between serum albumin (modeled as a continuous variable) and outcomes were examined using restricted cubic splines (RCS) with four knots placed at the 5th, 35th, 65th, and 95th percentiles. A serum albumin level of 32.9 g/L was used as the reference value. Nonlinearity was formally assessed using likelihood ratio tests. Additional subgroup analyses stratified by age, sex, and comorbidities were considered exploratory. All statistical analyses were conducted using R (version 4.5.1). A two-sided *P* value < 0.05 was considered statistically significant.

## Results

## Cohort characteristics

**Figure 1:**
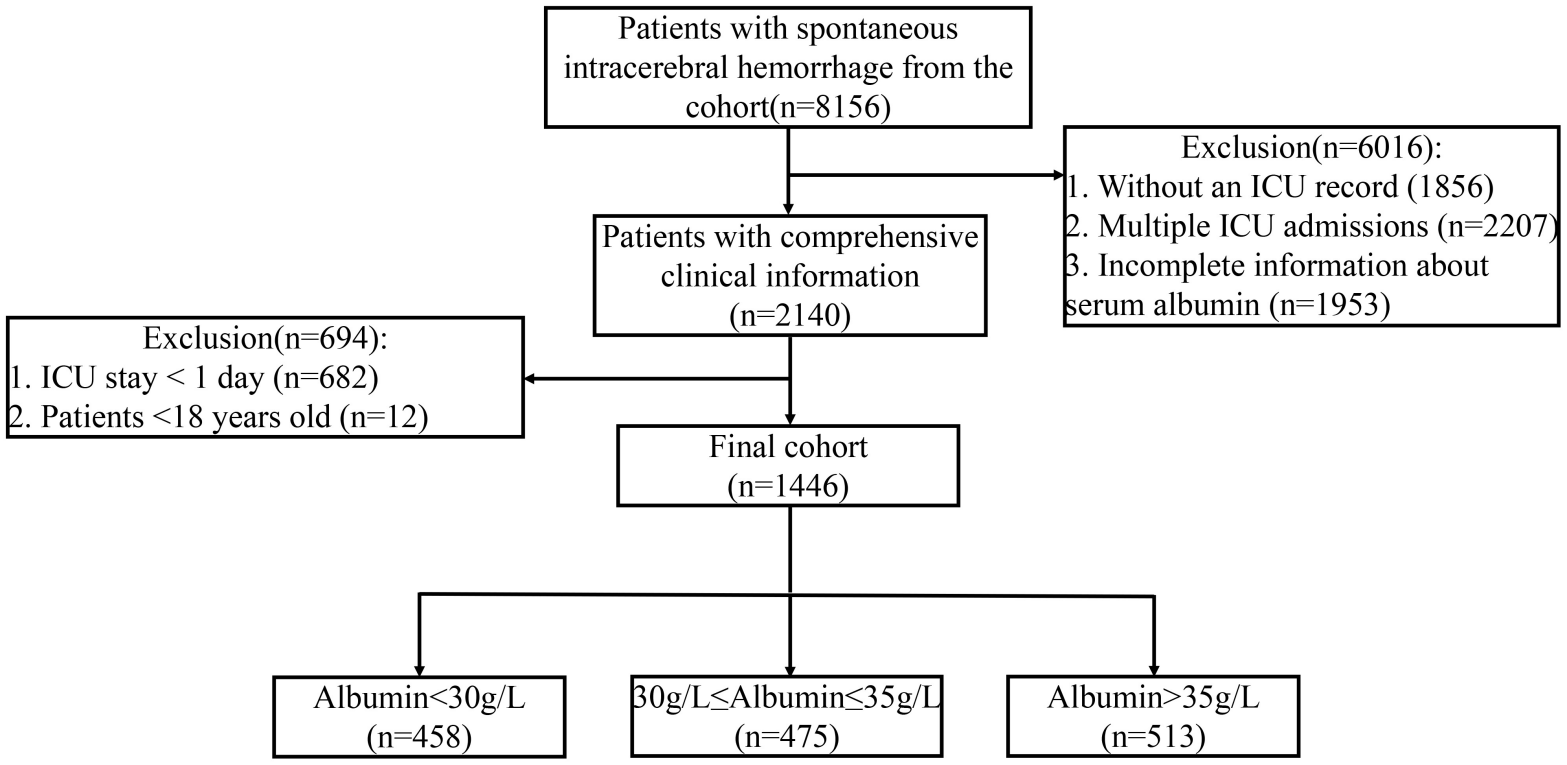
Flowchart of patient selection.

A total of 8,156 patients were initially identified from MIMIC-IV and the Tianjin cohort. After applying the predefined inclusion and exclusion criteria, 1,446 patients were included in the final pooled analytic cohort, and the patient selection process is illustrated in Figure 1. The mean age was 65.3 ± 15.7 years, and 781 (54.0%) patients were male. Participants were stratified into three groups according to clinically relevant thresholds of the lowest serum albumin level during hospitalization (>35 g/L, 30–35 g/L, and < 30 g/L; Table 1). Patients with lower serum albumin levels were more likely to have comorbid conditions, including myocardial infarction (*P* < 0.001), congestive heart failure (*P* < 0.001), and chronic pulmonary disease (*P* = 0.010), and had higher Charlson Comorbidity Index scores (*P* < 0.001). They also exhibited higher levels of WBC, blood glucose, and serum creatinine (all *P* < 0.001).

**Table 1:** Demographic and clinical characteristics at baseline according to albumin tertiles.

Characteristic	Total (*n* = 1446)	T1 (Albumin>35g/L) (*n* = 513)	T2 (30g/L ≤ Albumin ≤ 35g/L) (*n* = 475)	T3 (Albumin < 30g/L) (*n* = 458)	*P* value
Age, mean ± SD, y	65.3 ± 15.7	64.9 ± 15.9	67.0 ± 15.0	64.1 ± 16.0	0.014
Gender, *n* (%)					0.613
Female	665 (46.0)	228 (44.4)	226 (47.6)	211 (46.1)	
Male	781 (54.0)	285 (55.6)	249 (52.4)	247 (53.9)	
Race, *n* (%)					0.011
White	798 (55.2)	302 (58.9)	268 (56.4)	228 (49.8)	
Black	132 (9.1)	51 (9.9)	39 (8.2)	42 (9.2)	
Asian	45 (3.1)	21 (4.1)	12 (2.5)	12 (2.6)	
Other	471 (32.6)	139 (27.1)	156 (32.8)	176 (38.4)	
SBP, mean ± SD, mm Hg	127.1 ± 14.9	131 ± 13.3	128.0 ± 13.8	122.0 ± 16.1	< 0.001
DBP, mean ± SD, mm Hg	66.9 ± 10.9	69.5 ± 11.2	66.4 ± 10.9	64.3 ± 10.0	< 0.001
Laboratory measurements					
WBC, M(IQR), 10^9^/L	11.0 (8.3–14.7)	10.0 (7.7–13.0)	11.5 (8.5–14.9)	12.1 (9.3–16.2)	< 0.001
Hemoglobin, M(IQR), g/dL	11.9 (10.4–13.1)	12.6 (11.4–13.7)	11.8 (10.4–12.9)	11 (9.4–12.6)	< 0.001
Platelet, M(IQR), 10^9^/L	201.5 (152.0–262.1)	205.0 (167.0–259.0)	211.0 (160.0–267.0)	181.0 (118.0–259.0)	< 0.001
Creatinine, M(IQR), mg/dL	0.9 (0.7–1.2)	0.85 (0.7–1.1)	0.9 (0.7–1.2)	1.0 (0.7–1.5)	< 0.001
Blood Glucose, mmol/L	135.8 (113.4–170.0)	131.0 (112.0–158.0)	137.0 (116.0–172.0)	140.0 (114.0–176.0)	< 0.001
Comorbidities, *n* (%)					
Myocardial infarction	162 (11.2)	40 (7.8)	51 (10.7)	71 (15.5)	< 0.001
Congestive heart failure	212 (14.7)	45 (8.8)	74 (15.6)	93 (20.3)	< 0.001
Chronic pulmonary disease	201 (13.9)	53 (10.3)	71 (14.9)	77 (16.8)	0.010
Renal disease	189 (13.1)	49 (9.6)	60 (12.6)	80 (17.5)	0.001
Liver disease	148 (10.2)	24 (4.7)	44 (9.3)	80 (17.5)	< 0.001
Malignant cancer	124 (8.6)	46 (9.0)	37 (7.8)	41 (9.0)	0.757
Scoring systems					
GCS, M(IQR)	14 (10–15)	14 (12–15)	14 (10–15)	15 (10–15)	0.086
Charlson comorbidity index, M(IQR)	5 (3–7)	5 (3–7)	5 (4–7)	6 (4–8)	< 0.001
Mechanical ventilation, *n*(%)	747 (51.7)	155 (30.2)	274 (57.7)	318 (69.4)	< 0.001

SBP, systolic blood pressure; DBP, diastolic blood pressure; SD, standard deviation; WBC, white blood cell; IQR, interquartile range; GCS, Glasgow Coma Scale.

## Clinical outcomes of patients with different serum albumin levels

**Figure 2:**
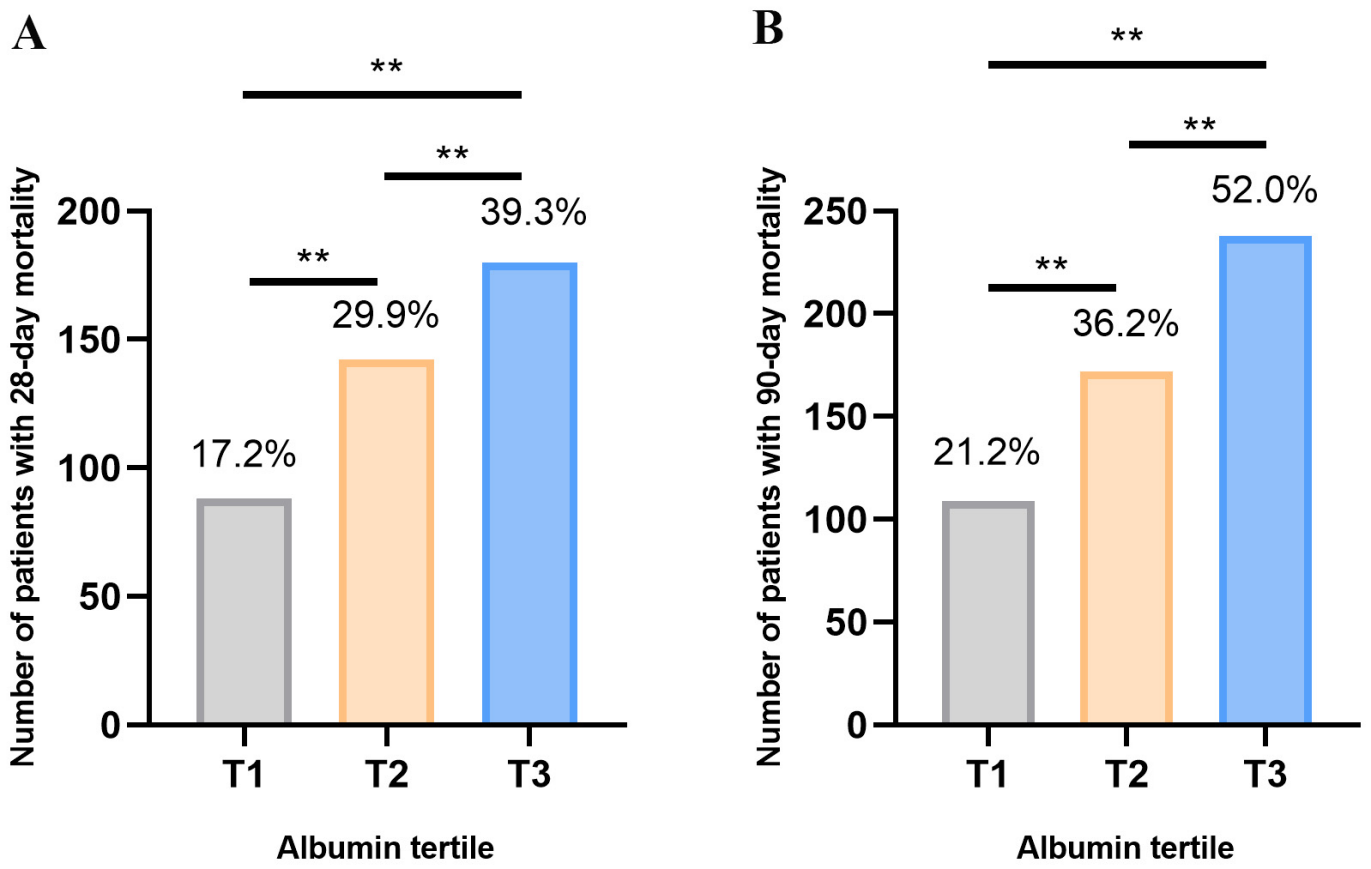
The incidence of 28-day mortality **(A)** and 90-day mortality **(B)** across serum albumin tertile groups. ^**^P < 0.01.

In-hospital mortality and length of hospital stay both increased significantly across groups (*P* for trend < 0.001). The median length of hospital stay was 11.78 days (interquartile range [IQR], 5.97–21.60), and the median ICU length of stay was 6.47 days (IQR, 2.80–12.83). The overall in-hospital mortality and ICU mortality rates were 25.9% and 18.2%, respectively (Table 2). The 28-day and 90-day mortality rates increased progressively across decreasing serum albumin categories. Specifically, 28-day mortality was 17.2%, 29.9%, and 39.3%, and 90-day mortality was 21.2%, 36.2%, and 52.0% among patients with albumin levels >35 g/L, 30–35 g/L, and < 30 g/L, respectively (*P* for trend < 0.001) (Table 2, Figure 2).

**Table 2:** Clinical outcomes of patients with different serum albumin levels.

Clinical outcome	Total (*n* = 1,446)	T1 (Albumin>35g/L) (*n* = 513)	T2 (30g/L ≤ Albumin ≤ 35g/L) (*n* = 475)	T3 (Albumin < 30g/L) (*n* = 458)	*P* value
Primary					
28–day mortality, *n* (%)	410 (28.4%)	88 (17.2%)	142 (29.9%)	180 (39.3%)	< 0.001
Secondary					
90–day mortality, *n* (%)	519 (35.9%)	109 (21.2%)	172 (36.2%)	238 (52.0%)	< 0.001
ICU mortality, *n* (%)	263 (18.2%)	51 (9.9%)	80 (16.8%)	132 (28.8%)	< 0.001
In–hospital mortality, *n* (%)	374 (25.9%)	73 (14.2%)	114 (24.0%)	187 (40.8%)	< 0.001
Length of ICU stay (days), median (IQR)	6.47 (2.80–12.83)	3.83 (1.98–7.22)	6.93 (2.84–12.60)	11.20 (4.80–20.00)	< 0.001
Length of hospital stay (days), median (IQR)	11.78 (5.97–21.60)	8.68 (4.61–13.80)	12.10 (6.57–20.90)	17.70 (9.63–29.90)	< 0.001

ICU, intensive care unit; IQR, interquartile range.

**Figure 3:**
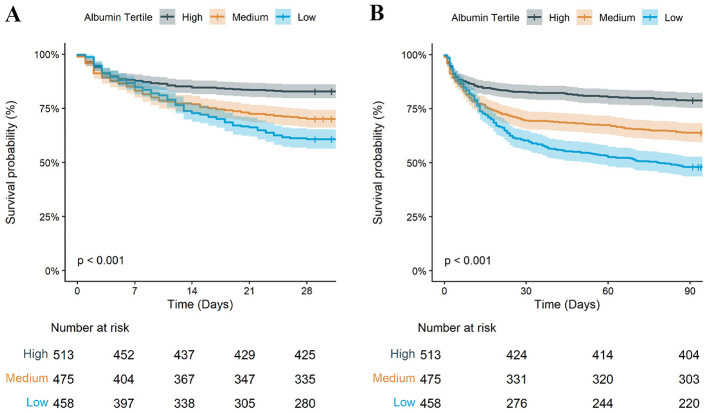
Kaplan-Meier survival curves for 28-day mortality **(A)** and 90-day mortality **(B)** by serum albumin tertile groups.

Kaplan-Meier survival curves for 28-day and 90-day mortality stratified by albumin categories are presented in Figure 3. Lower albumin levels were associated with a significantly higher risk of mortality at both time points (log-rank *P* < 0.001).

## Association between albumin and mortality outcomes in regression models

Regression models were used to evaluate the association between serum albumin and mortality outcomes (Table 3). In the fully adjusted model, lower serum albumin levels were consistently associated with increased risks of ICU mortality, in-hospital mortality, 28-day mortality, and 90-day mortality. Compared with patients with albumin >35 g/L, those with albumin < 30 g/L had significantly higher risks of ICU mortality (OR 2.51, 95% CI 1.68–3.76; *P* < 0.001), in-hospital mortality (OR 2.80, 95% CI 1.97–3.99; *P* < 0.001), 28-day mortality (HR 1.67, 95% CI 1.26–2.20; *P* = 0.0003), and 90-day mortality (HR 1.97, 95% CI 1.54–2.52; *P* < 0.0001).

**Table 3:** Regression analyses of the association between serum albumin and mortality outcomes.

Categories	Model 1		Model 2		Model 3	
	OR/HR (95% CI)	*p* value	OR/HR (95% CI)	*p* value	OR/HR (95% CI)	*p* value
ICU mortality						
per 1 g/L increase	0.93 (0.91–0.94)	< 0.001	0.93 (0.91–0.95)	< 0.001	0.94 (0.92–0.97)	< 0.001
Categorical trend (3–category)	1.93 (1.62–2.29)	< 0.001	1.87 (1.57–2.23)	< 0.001	1.60 (1.31–1.95)	< 0.001
T1	Reference	Reference	Reference	Reference	Reference	Reference
T2	1.84 (1.26–2.67)	0.002	1.78 (1.22–2.60)	0.003	1.46 (0.97–2.20)	0.068
T3	3.67 (2.58–5.22)	< 0.001	3.45 (2.42–4.93)	< 0.001	2.51 (1.68–3.76)	< 0.001
In–hospital mortality						
per 1 g/L increase	0.91 (0.90–0.93)	< 0.001	0.91 (0.90–0.93)	< 0.001	0.93 (0.91–0.95)	< 0.001
Categorical trend (3–category)	2.05 (1.76–2.40)	< 0.001	2.02 (1.73–2.37)	< 0.001	1.70 (1.42–2.02)	< 0.001
T1	Reference	Reference	Reference	Reference	Reference	Reference
T2	1.90 (1.38–2.64)	< 0.001	1.81 (1.30–2.52)	< 0.001	1.50 (1.05–2.13)	0.025
T3	4.16 (3.05–5.67)	< 0.001	4.02 (2.94–5.50)	< 0.001	2.80 (1.97–3.99)	< 0.001
28–day mortality						
per 1 g/L increase	0.95 (0.94–0.97)	< 0.001	0.95 (0.94–0.97)	< 0.001	0.97 (0.96–0.99)	< 0.001
Categorical trend (3–category)	1.54 (1.36–1.74)	< 0.001	1.50 (1.32–1.69)	< 0.001	1.28 (1.12–1.46)	< 0.001
T1	Reference	Reference	Reference	Reference	Reference	Reference
T2	1.86 (1.42–2.42)	< 0.001	1.71 (1.31–2.23)	< 0.001	1.42 (1.08–1.86)	0.013
T3	2.46 (1.91–3.18)	< 0.001	2.31 (1.79–2.98)	< 0.001	1.67 (1.26–2.20)	< 0.001
90–day mortality						
per 1 g/L increase	0.94 (0.93–0.96)	< 0.001	0.94 (0.93–0.96)	< 0.001	0.96 (0.95–0.98)	< 0.001
Categorical trend (3–category)	1.66 (1.49–1.85)	< 0.001	1.64 (1.46–1.83)	< 0.001	1.40 (1.24–1.58)	< 0.001
T1	Reference	Reference	Reference	Reference	Reference	Reference
T2	1.87 (1.47–2.38)	< 0.001	1.72 (1.35–2.19)	< 0.001	1.43 (1.11–1.83)	0.005
T3	2.82 (2.25–3.54)	< 0.001	2.71 (2.15–3.40)	< 0.001	1.97 (1.54–2.52)	< 0.001

HR, hazard ratio; CI, confidence interval.

Model 1: unadjusted.

Model 2: adjusted for age, sex, race.

Model 3: adjusted for age, gender, race, sbp, dbp, wbc, hemoglobin, platelet, creatinine, blood glucose, myocardial infarct, congestive heart failure, chronic pulmonary disease, renal disease, liver disease, malignant cancer, charlson comorbidity index, GCS.

ORs were estimated using logistic regression models for ICU mortality and In-hospital mortality. HRs were estimated using Cox proportional hazards regression models for 28-day and 90-day mortality. All estimates are presented with 95% CIs.

**Figure 4:**
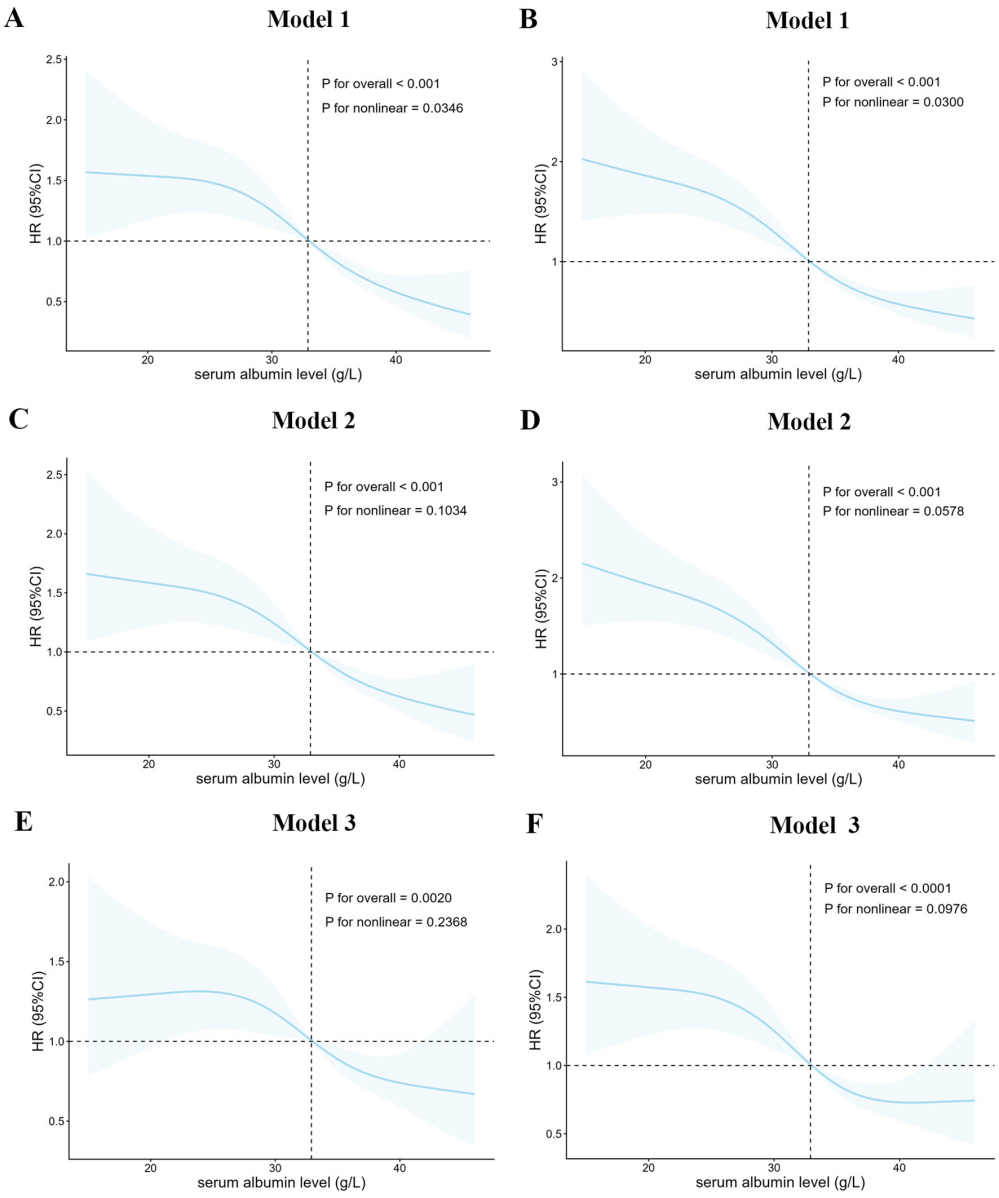
RCS curves of serum albumin with the mortality. **(A, C**, and **E)** 28-day mortality; **(B, D**, and **F)** 90-day mortality.

A significant graded association was observed across albumin categories for all outcomes (all *P* for trend < 0.001). When modeled as a continuous variable, each 1 g/L increase in serum albumin was associated with a reduced risk of mortality across all endpoints. RCS analyses were performed to examine potential nonlinear associations between continuous serum albumin levels and mortality outcomes. The corresponding *P* values for overall association and nonlinearity are presented in Figure 4. Lower albumin levels were associated with higher mortality risk, with no strong evidence of nonlinearity observed in the fully adjusted models.

## Subgroup analyses by mechanical ventilation status

**Figure 5:**
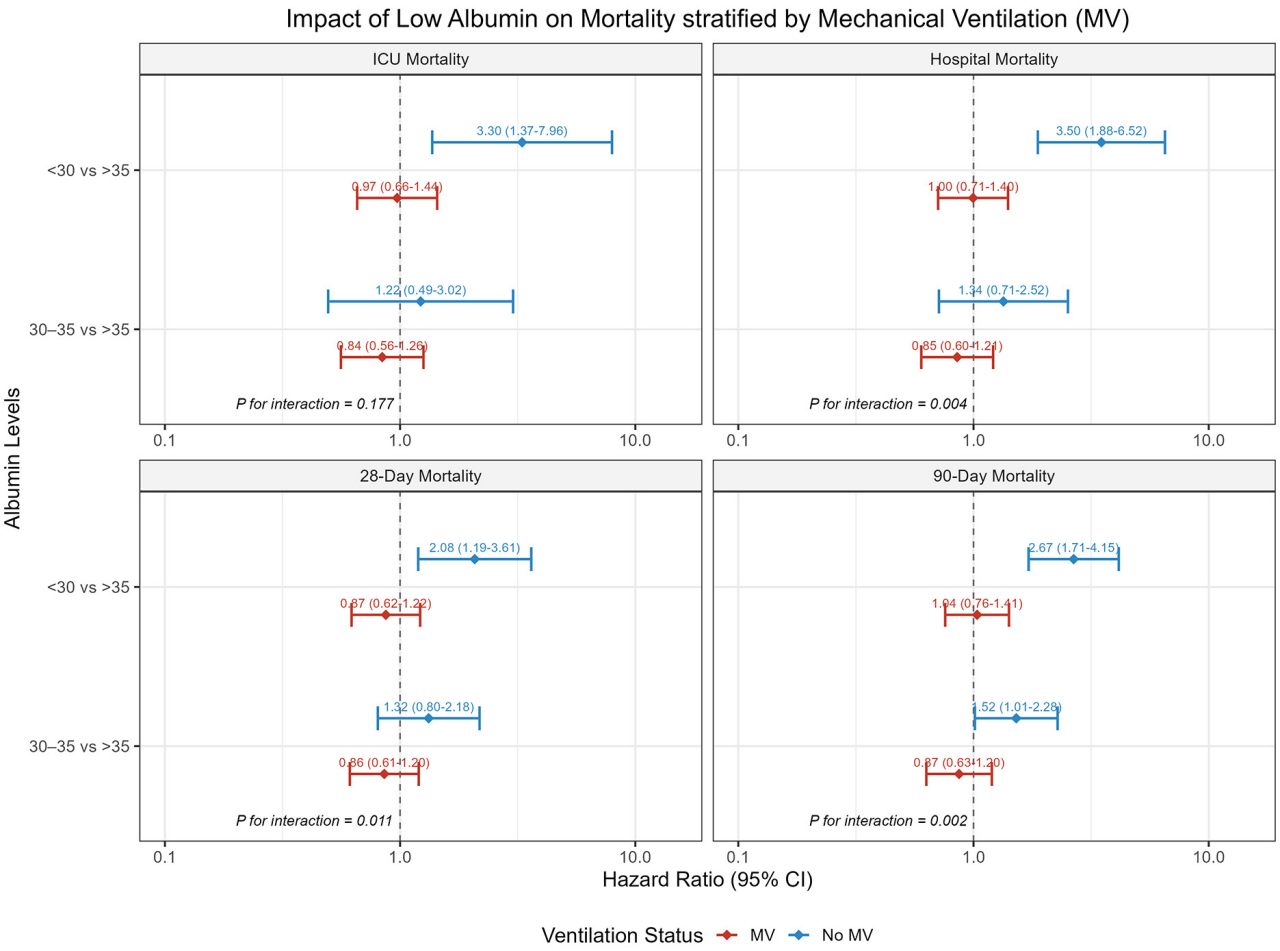
Hazard ratios (HR) for mortality outcomes (ICU, hospital, 28-day, and 90-day) stratified by mechanical ventilation (MV) and albumin levels.

Given that MV may reflect both disease severity and treatment intensity, we further evaluated whether the association between serum albumin and mortality varied by MV status. In stratified analyses, lower serum albumin levels were strongly associated with increased mortality among patients not receiving MV. For instance, compared with albumin >35 g/L, albumin < 30 g/L was associated with higher risks of ICU mortality (HR 3.30, 95% CI 1.37–7.96), in-hospital mortality (HR 3.50, 95% CI 1.88–6.52), 28-day mortality (HR 2.08, 95% CI 1.19–3.61), and 90-day mortality (HR 2.67, 95% CI 1.71–4.15). In contrast, these associations were attenuated among patients receiving MV (Figure 5). Formal interaction analyses demonstrated significant effect modification by MV status for in-hospital mortality (*P* for interaction = 0.0042), 28-day mortality (*P* = 0.0113), and 90-day mortality (*P* = 0.0024), but not for ICU mortality (*P* = 0.1771).

**Figure 6:**
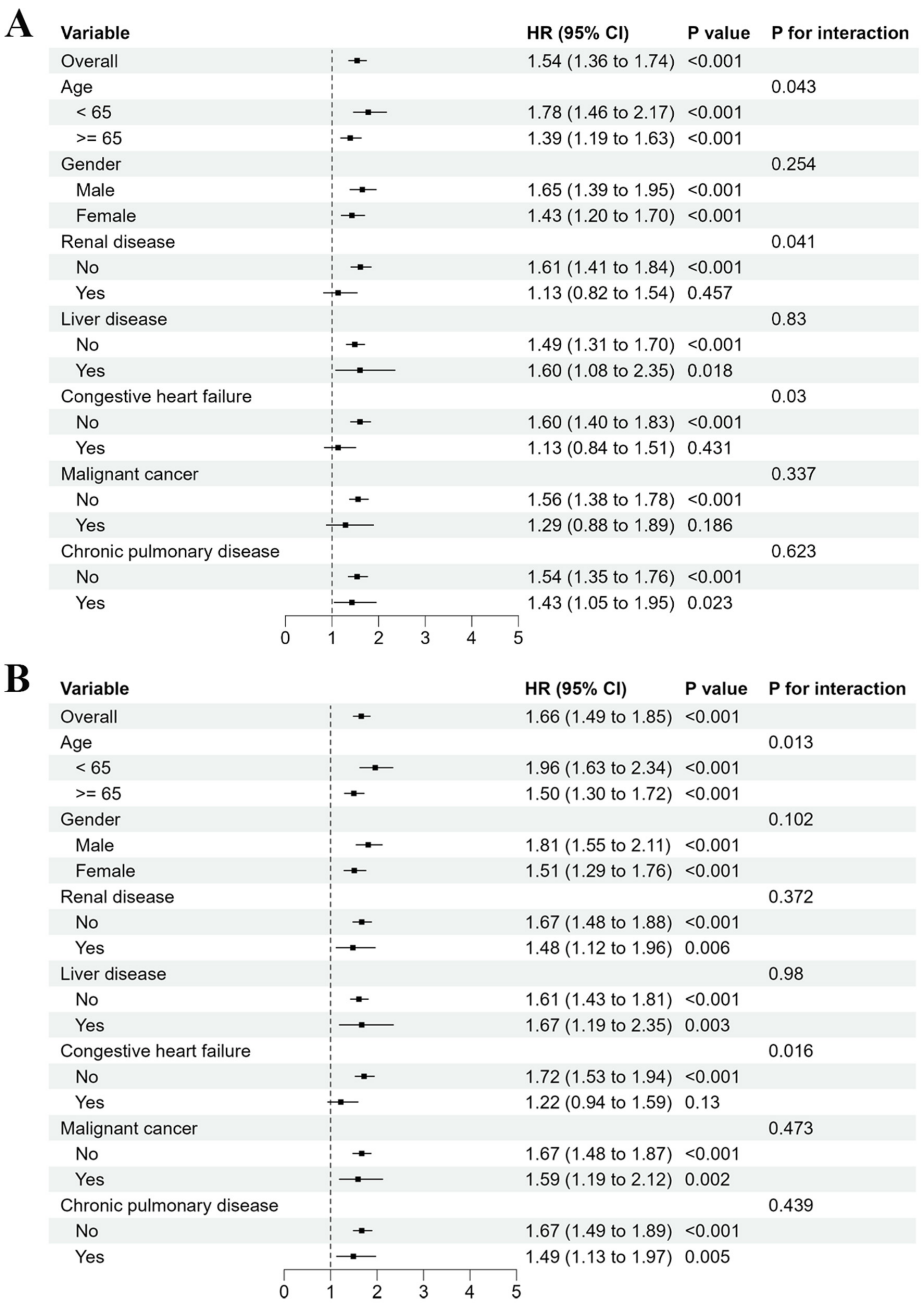
Subgroup analysis—Forest plot of 28-day mortality **(A)** and 90-day mortality **(B)**.

Additional subgroup analyses stratified by age, sex, and comorbidities are presented in Figure 6 and should be interpreted as exploratory.

## Discussion

In this cohort study, lower nadir serum albumin levels during hospitalization were associated with increased mortality among patients with spontaneous ICH. After adjustment for a comprehensive set of clinically relevant covariates, the lowest serum albumin level remained independently associated with both short-term (28-day) and longer-term (90-day) mortality. We further observed significant effect modification by MV status. The association between hypoalbuminemia and mortality was evident among patients not receiving MV but was substantially attenuated among those undergoing MV. These findings suggest that the prognostic value of serum albumin may vary across different levels of illness severity and treatment intensity.

Our findings are consistent with prior evidence linking hypoalbuminemia to adverse outcomes in critically ill populations. For example, Jin et al. (9) reported that serum albumin levels < 30 g/L were independently associated with increased ICU and in-hospital mortality in a large cohort of critically ill patients. Similar associations have been observed in pediatric ICU populations and in patients with severe sepsis, where persistently low albumin trajectories were associated with the highest mortality risk (11, 15). In neurologic populations, admission hypoalbuminemia (defined as serum albumin ≤ 35 g/L) has also been associated with increased 90-day mortality following acute ICH (16). Extending these observations, our study highlights the prognostic value of the lowest serum albumin level during hospitalization in patients with spontaneous ICH.

Several biological pathways may plausibly link low albumin to worse outcomes after ICH. Albumin has antioxidant, antiapoptotic, and neuroprotective properties (17–20), which may be relevant to secondary brain injury processes in ICH, including oxidative stress, blood-brain barrier disruption, and immune activation (1, 4). Albumin also contributes to plasma oncotic pressure (21); reduced levels may exacerbate capillary leak and tissue edema (22), potentially aggravating cerebral edema, an important driver of neurological deterioration and mortality. In addition, albumin is a negative acute-phase reactant and may reflect a composite of systemic inflammation, hemodilution, hepatic dysfunction, and limited nutritional reserves (23). Thus, nadir albumin likely captures multiple dimensions of critical illness that are relevant to prognosis.

The strong association we observed aligns with accumulating evidence that highlights the prognostic utility of both absolute serum albumin levels and albumin-based indices in critically ill patients. For example, in emergency-department sepsis cohorts, each 1 g/dl decrease in albumin has been associated with a markedly higher risk of short-term mortality (12). Lowest serum albumin levels and declining serum albumin trends have been reported as potential predictors of mortality in ICU patients with sepsis (13). Albumin has also been incorporated into models predicting new-onset organ dysfunction after ICU admission (24) and has been linked to acute kidney injury in critical illness (14). Beyond albumin alone, composite measures such as the blood urea nitrogen-to-albumin ratio (BAR) have been used to identify high-risk acute kidney injury (AKI) patients (25) and to predict outcomes among ICU-treated acute ischemic stroke patients (26). In neurocritical care, the fibrinogen-to-albumin ratio has been reported as an independent predictor of hematoma expansion and in-hospital mortality in ICH (27, 28). Together, these findings—combined with the strong association of nadir albumin with longer-term mortality observed in our study—reinforce the clinical value of serial albumin monitoring and related measures as readily available markers for risk enrichment. Such approaches may help identify high-risk patients earlier and guide supportive care (e.g., nutritional assessment and fluid management), although prospective studies are needed to determine whether albumin-directed interventions improve outcomes.

Whether albumin represents a modifiable therapeutic target remains uncertain and appears to be highly context dependent. Evidence from clinical studies suggests that the benefit of albumin may depend on patient selection, timing, treatment duration, and therapeutic intent. In the SAFE trial, albumin was not superior to saline for general fluid resuscitation in ICU patients (29), and in the ALBIOS trial, albumin supplementation targeting a serum albumin concentration of at least 30 g/L did not improve mortality in the overall population of patients with severe sepsis or septic shock (10). Similarly, the ATTIRE trial showed no benefit of targeted albumin infusion in hospitalized patients with decompensated cirrhosis (30). In contrast, the ANSWER trial suggested that long-term scheduled albumin administration may improve survival in selected patients with decompensated cirrhosis and ascites (31). A recent retrospective analysis of the MIMIC-IV database further suggested that early, but not delayed, albumin administration combined with crystalloids during high-volume resuscitation was associated with lower 28-day and 60-day mortality (32). These findings suggest that the clinical benefit of albumin may vary substantially across different clinical contexts. Therefore, although nadir serum albumin may be useful for risk stratification, whether albumin correction can serve as a modifiable therapeutic strategy in ICU patients with spontaneous ICH requires confirmation in prospective randomized trials.

A key observation in our study is that the prognostic signal of albumin differed according to MV status. Few studies have examined whether life-support interventions influence the prognostic utility of albumin. Rather than indicating a causal interaction, MV status may identify populations with differing baseline severity and treatment intensity, in whom the relative prognostic contribution of albumin may differ. In ventilated patients, prognosis may be dominated by the underlying indication for MV, the severity of neurologic injury, and the broader intensity of organ support, which could attenuate the incremental prognostic information conveyed by albumin. In contrast, among non-ventilated patients who may have relatively lower baseline severity or greater physiologic stability, albumin may better reflect systemic inflammation and physiologic reserve. Therefore, this heterogeneity should be interpreted cautiously as differential prognostic value across clinically distinct severity strata, rather than as definitive evidence of causal effect modification by MV status.

In exploratory subgroup analyses, the association appeared more pronounced in younger patients and in those without renal disease. In patients with a substantial comorbidity burden, competing risks and dominant organ-failure pathways may overshadow the incremental prognostic contribution of nutritional-inflammatory markers (33). Conversely, in younger or less comorbid patients, hypoalbuminemia may more directly reflect heightened inflammatory stress and reduced physiologic reserve, thereby exhibiting a stronger association with adverse outcomes. Similar age-dependent prognostic patterns have been reported for other metabolic markers, such as the TyG index (34, 35).

Of course, there are still some limitations to our study. First, due to the retrospective study design, causality cannot be firmly inferred. While multivariable adjustments and subgroup analyses were undertaken, residual confounders may nonetheless have impacted the clinical results. Second, important neuroimaging and disease-specific severity variables, including hematoma volume, hematoma location, intraventricular hemorrhage, hydrocephalus, neurosurgical intervention, ICH score, and anticoagulant use, were unavailable or incompletely captured in the databases. Therefore, residual confounding related to baseline ICH severity cannot be excluded. Third, because nadir serum albumin during hospitalization is a time-varying exposure, the main analysis may be affected by immortal time bias. Patients who survive longer may have more opportunities to develop lower albumin levels, whereas patients who die early have a shorter observation window. Therefore, the observed association should be interpreted as prognostic rather than causal. Future studies using fixed early-window albumin measurements or time-dependent Cox models are needed to better evaluate the temporal relationship between albumin changes and mortality. Fourth, patients from MIMIC-IV and the Tianjin cohort were combined for analysis, and the Tianjin cohort was not used as an independent external validation cohort; therefore, potential heterogeneity between data sources may remain.

## Conclusions

Lower nadir serum albumin levels during hospitalization were independently associated with increased short- and long-term mortality in patients with spontaneous ICH. The association between nadir serum albumin and mortality appeared to differ across MV strata, with a stronger signal among non-ventilated patients and an attenuated association among ventilated patients. These findings support nadir serum albumin as a readily available marker for risk stratification, particularly for identifying higher-risk patients within the non-ventilated subgroup.

## Data Availability

The raw data supporting the conclusions of this article will be made available by the authors, without undue reservation.
